# Bioengineering Lantibiotics for Therapeutic Success

**DOI:** 10.3389/fmicb.2015.01363

**Published:** 2015-11-27

**Authors:** Des Field, Paul D. Cotter, Colin Hill, R. P. Ross

**Affiliations:** ^1^School of Microbiology, University College Cork, Cork, Ireland; ^2^Teagasc Food Research Centre, Fermoy, Ireland; ^3^APC Microbiome Institute, University College Cork, Cork, Ireland

**Keywords:** antimicrobial peptide, nisin, mutagenesis, lantibiotic, post-translational modification, bacteriocin, bacterial resistance

## Abstract

Several examples of highly modified antimicrobial peptides have been described. While many such peptides are non-ribosomally synthesized, ribosomally synthesized equivalents are being discovered with increased frequency. Of the latter group, the lantibiotics continue to attract most attention. In the present review, we discuss the implementation of *in vivo* and *in vitro* engineering systems to alter, and even enhance, the antimicrobial activity, antibacterial spectrum and physico-chemical properties, including heat stability, solubility, diffusion and protease resistance, of these compounds. Additionally, we discuss the potential applications of these lantibiotics for use as therapeutics.

## Introduction

Given that antibiotic resistance has now reached a crisis point, novel compounds and innovative methods are urgently required to arrest the spread and development of drug-resistant infections in both the nosocomial and community environments. Ideally, such novel substances should exhibit distinctly different mechanisms of action to currently used chemotherapeutics in order to decrease resistance development. Ribosomally synthesized antimicrobial peptides produced by bacteria (bacteriocins) constitute an emerging class of natural products that have attracted considerable interest as promising alternatives to existing antibiotics (Sahl and Bierbaum, 2008). Within this diverse group of peptides, the lantibiotics, i.e., class I bacteriocins which contain the post-translationally modified amino acids lanthionine and methyllanthionine, have become the focus of many biomedical and pharmaceutical research groups due to their demonstrable high potency *in vitro*, multiple modes of action and ability to destroy target cells rapidly (Cotter et al., 2005; Cavera et al., 2015). In general, lantibiotics exhibit activity against Gram positive bacteria. Importantly, this includes many drug resistant targets including methicillin resistant *Staphylococcus aureus* (MRSA), vancomycin intermediate *S. aureus* (VISA), vancomycin resistant enterococci (VRE), *Streptococcus pneumoniae* and *Clostridium difficile*, amongst others (Cotter et al., 2013). Furthermore, several lantibiotic peptides have demonstrated excellent *in vivo* activities and have progressed toward clinical evaluation for the treatment of life-threatening diseases (Dawson and Scott, 2012; Sandiford, 2015). Indeed, these and a range of other desirable features make them suitable for use in human and veterinary medicine and also in the pharmaceutical industry (Dischinger et al., 2014). However, despite these promising attributes, there are a number of limitations that has prevented their more widespread use, including instability and/or insolubility at physiological pH, low production levels and susceptibility to proteolytic digestion. The implementation of multiple technologies, including genome mining as well as high-throughput screening strategies in combination with *in vivo* and *in vitro* expression systems has provided a wealth of information relating to the widespread existence, structural diversity and functionality of lantibiotics while facilitating the identification of structural regions that can be targeted to enhance their biological and physicochemical properties. The present review will focus on recent developments with regard to these achievements.

## Lantibiotics: The Case for Therapeutic Use (*in vitro* and *in vivo* Potency)

New antimicrobials that possess novel modes of action, particularly against drug resistant organisms so that they can be specifically targeted for clinical applications, are required as a matter of urgency. In this regard lantibiotics hold considerable potential as a consequence of their unusual structure, unique mechanisms of action and their potency against multi-drug resistant bacteria. Today, close to 100 of these bioactive peptides have been described, the majority of which are produced by Gram-positive bacteria (Dischinger et al., 2014). The common feature that links all lantibiotics is the presence of a number of distinctive amino acids which result from enzymatically mediated post-translational modifications, including dehydration and cyclisation, leading to the formation of the eponymous (methyl)lanthionine bridges. These bridges convert the linear peptide chain into a polycyclic form giving structure and function to the peptide. It should be noted that only those peptides that display antimicrobial activity within the larger family of lanthionine-containing peptides or lanthipeptides are termed lantibiotics.

**FIGURE 1:**
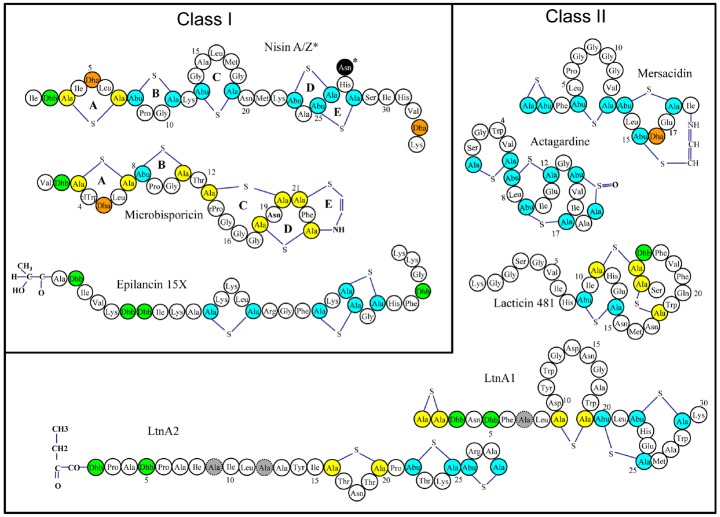
**Representative structures of various single and two-component lantibiotics.** Post translational modifications are indicated as follows: Abu: 2-aminobutyric acid, Ala-S-Ala: lanthionine, Abu-S-Ala: 3-methyllanthionine, Dha: dehydroalanine, Dhb: dehydrobutyrine. D-Ala residues (in lacticin 3147) are shaded gray.

Many lantibiotics exert their antimicrobial action through complexation with lipid II, an essential precursor of the bacterial cell wall, either by inhibiting cell wall synthesis through sequestration of lipid II and/or by disruption of membrane integrity and pore formation (Breukink and de Kruijff, 2006). Indeed, the prototypical and best studied lantibiotic nisin performs both of these functions as a consequence of two distinct structural domains located at the N- and C-termini (Figure 1). It has been established that the A, B, and C rings form a “cage-like” enclosure that facilitates binding of the pyrophosphate moiety of lipid II, thus inhibiting cell wall synthesis (Hsu et al., 2004) This binding enhances the ability of the C-terminal segment, containing rings D and E, to form pores in the cell membrane, resulting in the rapid efflux of ions and cytoplasmic solutes (Wiedemann et al., 2001). This mechanism of action is not common to all lantibiotics, and some of them lack the ability to elicit pores or to bind lipid II or both, but can still exhibit antimicrobial activity (Pag and Sahl, 2002). The poor activity of lantibiotics toward Gram negative bacteria is due to the outer membrane (OM) of the Gram negative cell wall which acts as a barrier for the cell, restricting the access of the peptides to the cytoplasmic membrane (Nikaido and Vaara, 1985).

Lantibiotics have been classified on the basis of their biosynthetic pathways (Willey and van der Donk, 2007). According to this scheme, class I lantibiotics are those modified by two separate enzymes, a LanB (dehydratase) and LanC (cyclase); class II are modified by a single LanM enzyme with both dehydratase and cyclase activity. The third and fourth classes of lanthipeptides are also modified by a single enzyme (general nomenclature LanKC for class III and LanL for class IV; van der Donk and Nair, 2014). Most of the class III lanthipeptides reported thus far have no or weak antimicrobial activities, but some have been shown to possess anti-allodynic/antinociceptive activity (Meindl et al., 2010; Iorio et al., 2014), antiviral activity (Férir et al., 2013) or morphogenetic activities (Willey and Gaskell, 2011). The designation Lan is used generically to refer to proteins associated with the biosynthesis of, or immunity to, lantibiotics. A typical lantibiotic operon will also contain genes encoding enzymes to carry out transport/processing (LanT), immunity (LanI and LanFEG), proteolytic processing (LanP) as well as the structural gene (LanA). Other enzymes, responsible for the formation of less common residues may also be present. Importantly, individual components of the lantibiotic biosynthetic machinery show even greater flexibility as demonstrated by their activity *in vitro* (Li et al., 2009).

## Activity of Lantibiotics *in vitro*

Although lantibiotics such as nisin have been in use for decades as safe and natural food preservatives (Delves-Broughton, 2005), the continued escalation of multi-drug resistant bacterial infections has led to a re-appraisal of their capacity for use against life-threatening infections. A multitude of studies have highlighted the *in vitro* potency of lantibiotics against nosocomial pathogens (the reader is directed to a comprehensive review: Piper et al., 2009a). Many lantibiotics, including lacticin 3147, mutacins B-Ny266 and 1140, nisin, mersacidin, epidermin, Pep5, and planosporicin exhibit activity against clinically-relevant targets (Table 1) such as MRSA, VRE, *Propionibacterium acne*, *Streptococcus mutans*, *Streptococcus pyogenes*, *S. pneumoniae*, *C. difficile*, *Listeria*, and *Bacillus* species (Severina et al., 1998; Galvin et al., 1999; Mota-Meira et al., 2000; Brumfitt et al., 2002; Rea et al., 2007; Ghobrial et al., 2009; Piper et al., 2009b). Notably, both Pep 5 and epidermin successfully inhibit the adhesion of staphylococcal cells to the surfaces of siliconized catheters (Fontana et al., 2006). Although it is the general view that lantibiotics exhibit less potential as chemotherapeutics to combat infections with Gram-negative organisms, lantibiotics including mutacin B-Ny266 are selectively active against a few strains of *Neisseria* and *Helicobacter* (Mota-Meira et al., 2000), while purified nisin displays activity against *Escherichia coli* (Kuwano et al., 2005).

**TABLE 1:** **A Selection of Lantibiotics and their Potential Therapeutic Applications**.

**Lantibiotic**	**Commercially relevant targets**	***In vivo* tests**	**Potential applications**	**Reference**
Nisin	Gram positive bacteria	✓	Treatment of staphylococcal (including MRSA) and enterococcal infections. Treatment of bacterial mastitis. Oral hygiene, deodorants. Anti-cancer	Mota-Meira et al., 2000; Brumfitt et al. (2002); Cotter et al. (2005); Piper et al., 2009b; Joo et al. (2012); Kamarajan et al. (2015)
Mersacidin	MRSA VRE, *C. difficile*	✓	Treatment of staphylococcal (including MRSA) and enterococcal infections. Treatment of CDAD	Niu and Neu (1991); Hoffmann et al. (2002); Appleyard et al. (2009)
Actagardine	MRSA, VRE, *C. difficile*	✓	Treatment of staphylococcal (including MRSA) and enterococcal infections. Treatment of CDAD	Hoffmann et al. (2002)
Deoxyactagardine/NVB302	*C. difficile*	✓	Treatment of *C. difficile* infections	Dawson and Scott (2012)
Gallidermin/Epidermin	*Propionibacteria*, Staphylococci, Streptococci	✓	Skin disorders including acne, eczema, folliculitis and impetigo	Bonelli et al. (2006)
Pinensins	Yeast/fungi	✓	Antifungal/yeast	Mohr et al. (2015)
Planosporicin	MRSA, VRE, Streptococci	✓	Treatment of staphylococcal (including MRSA) and enterococcal infections including VRE	Castiglione et al. (2007)
Microbisporicin	MRSA, VISA, VRE, *C. difficile*	✓	Treatment of staphylococcal (including MRSA and VISA) and enterococcal infections including VRE. Acne	Castiglione et al. (2008)
Mutacin B-Ny266	Multi-drug resistant bacteria	✓	Treatment of multi-drug resistant bacteria including MRSA and VRE	Mota-Meira et al., 2000
Lacticin 3147	Gram positive bacteria	✓	Treatment of bacterial mastitis. staphylococcal and enterococcal infections including VRE. Acne	Galvin et al. (1999); Lawton et al. (2007); Piper et al. (2009b)
Salivaricin B	Streptococci including *S. pyogenes* and *S. sobrinus*	✓	Treatment of streptococcal infections with emphasis on the causative agents of sore throats (caused mainly by *S. pyogenes*) and dental caries (caused in part by *S. sobrinus*).	Tagg (2004); Wescombe et al. (2009)
Duramycin	Increase chloride transport and fluid secretions	✓	Treatment of Cystic Fibrosis, ocular diseases and disorders	Grasemann et al. (2007); Oliynyk et al. (2010)

Nisin has also been shown to effectively inhibit spore outgrowth including spores of *Bacillus anthracis* (Gut et al., 2008) and those of *C. difficile* (Nerandzic and Donskey, 2010). Additionally, studies have revealed that the lantibiotic gallidermin efficiently prevents biofilm formation in both *S. aureus* and *S. epidermidis* species (Saising et al., 2012).

Recently, an intriguing and novel (sub-)class of lantibiotics termed pinensins were found to be highly active against many filamentous fungi and yeasts but displayed only weak antibacterial activity. Not only do pinensin A and pinensin B represent the first examples of a lantibiotic fungicide, they are also the first lantibiotics to be isolated from a Gram-negative native producer (Mohr et al., 2015).

## Lantibiotics Demonstrate *in vivo* Potency

While the *in vitro* success of a chemotherapeutic agent does not always necessarily translate to *in vivo* efficacy, there have been a number of encouraging studies to suggest that this may not be a major shortcoming of lantibiotics. For instance, mutacin B-Ny266 was shown to be as active as vancomycin against MRSA *in vivo* (Mota-Meira et al., 2005), mersacidin was able to effectively eradicate an MRSA infection in a mouse rhinitis model (Kruszewska et al., 2004) and Nisin F, a natural nisin variant, was also found to successfully control *S. aureus* infection in rats (De Kwaadsteniet et al., 2009). Similarly, microbsporicin (Figure 1) (NAI-107) was evaluated for its therapeutic potential in nosocomial infection and demonstrated efficacy against MRSA in a rat endocarditis model (Jabes et al., 2011). The efficacy of MU1140 (mutacin 1140) has also been investigated *in vivo* (Ghobrial et al., 2009) and is currently in pre-clinical development for the treatment of Gram positive infections. NVB302, a derivative of deoxyactagardine B, is currently undergoing phase I clinical trials as a therapeutic for the treatment of *C. difficile* infections due to its selective targeting of this organism over the predominantly Gram negative normal gut flora (Dawson and Scott, 2012). Investigations into the use of lantibiotics to control the microorganisms responsible for dental plaque, halitosis and “sore throat” infections have also yielded promising results (Hillman, 2002; Burton et al., 2006; Dierksen et al., 2007).

Some lantibiotics possess additional bioactivities that hold promise for therapeutic application. A smaller subcategory of lantibiotics, such as cinnamycin and duramycin, have been found to influence eukaryotic metabolic functions by binding phosphatidylethanolamine in cell membranes and, in turn, inhibiting the enzyme phospholipase A2 (Marki et al., 1991). In addition to this activity, duramycin demonstrated efficacy in the treatment of cystic fibrosis by inhalation (Grasemann et al., 2007) as a result of its ability to stimulate chloride secretion in bronchial epithelial cells (Oliynyk et al., 2010).

Remarkably, the first instance of a lantibiotic, or indeed any bacteriocin, to prevent the growth of cancer cells has been confirmed. In a study by Joo and coworkers, nisin Z was shown to be effective in the treatment of head and neck squamous cell carcinoma (HNSCC; Joo et al., 2012). In subsequent mouse trials involving a highly purified form of nisin Z, reduced tumorigenesis *in vivo* was observed and long-term treatment with nisin Z extended survival. In addition, nisin treated mice exhibited normal organ histology with no evidence of inflammation, fibrosis or necrosis (Kamarajan et al., 2015).

## Bioengineering and Synthetic Biology- Generating More Effective Lantibiotics

Bioengineering (engineering within the cell) and the use of synthetic biology-based (*in vitro* engineering) approaches have been important for advancing our understanding of the fundamentals of bacteriocin activity and structure–function relationships (these approaches are the subject of a number of recent comprehensive reviews: Tabor, 2014; Escano and Smith, 2015). However, there is also a steadily growing number of engineered lantibiotic peptides that demonstrate enhanced functionalities (activity and/or stability) which make them more attractive from a clinical perspective (Cotter et al., 2013). The following provides some recent examples of bioengineered lantibiotics exhibiting enhanced pharmacological and physicochemical properties as well as developments in genetic systems to increase peptide yields.

Several bioengineered variants of the prototypical lantibiotic nisin have been generated that provide excellent examples of how lantibiotic functionality can be modulated by as little as one residue change. The nisin Z derivatives N20K, M21K, N27K, H31K generated by protein engineering displayed improved solubility, particularly at alkaline pH values where the solubility of the parent nisin is particularly reduced (Rollema et al., 1995; Yuan et al., 2004). Furthermore, the consequences of effecting single residue alterations at distinct locations in nisin has generated variants that exhibit not only improved antimicrobial activity against strains of clinical relevance (MRSA, VRE, VISA, MRSP, and *C. difficile*) but has also brought about the widening of its antimicrobial spectrum to include some Gram negative bacteria (Field et al., 2008, 2012, 2015; Molloy et al., 2013). More dramatic substitutions at the location of rings A and B at the N-terminal end of nisin A revealed that the various activities of nisin can be altered by changing the amino acid arrangement in this region of the peptide (Rink et al., 2007). The hinge-region of nisin has also been the subject of mutagenesis resulting in variants with enhanced antimicrobial activity (Field et al., 2008; Healy et al., 2013) as well as derivatives with an enhanced ability to diffuse through complex polymers (Rouse et al., 2012). In both mutacin 1140 and nukacin ISK-1 peptides, single residue changes brought about a significant increase in activity against several Gram positive strains(Islam et al., 2009; Chen et al., 2013). Similarly, mutagenesis of the mersacidin gene was ultimately successful in that several variants were identified which exhibited enhanced activity against a range of different targets including clinically relevant MRSA, VRE and *S. pneumoniae* (Appleyard et al., 2009).

Generating enhanced variants of two-component lantibiotics presents an even greater challenge given that two peptides are required to work jointly in synergy. However, a lacticin 3147 derivative with enhanced activity against a pathogenic strain of *S. aureus* was recently identified (Field et al., 2013), the first occasion such an increase in antibacterial properties has been observed for bioengineered two-component lantibiotics.

Synthetic biology approaches are another promising means to provide insights into structure-stability relationships and generate novel derivatives with improved function. Chemical synthesis enables the limitations of the modification machinery to be bypassed, extending the range of analogs that can be produced. For example, deoxyactagardine B is a single peptide lantibiotic that is rigid, compact and globular and differs from actagardine (Figure 1) by two amino acids and the absence of a sulfoxide bond (Boakes et al., 2010). A synthetically introduced C-terminal modification (1, 7 diaminoheptane) produced a variant, NVB302, that displayed greater solubility and activity compared to the parent molecule. NVB302 is now in phase I clinical trials for the treatment of *C. difficile* infections (Dawson and Scott, 2012). It has also been established that lantibiotics are susceptible to oxidation of the sulfur-containing lanthionine and this can lead to sharp decreases in antimicrobial activity. In the case of lactocin S, lanthionines were replaced with diaminopimelate to produce several analogs, one of which revealed greater stability whilst still retaining 100% biological activity (Ross et al., 2012).

Chemical synthesis methods were employed to produce enhanced analogs of the lantibiotic epilancin 15X (Knerr and van der Donk, 2012). A novel approach termed *in vitro* mutasynthesis has produced improved variants of the class II lantibiotic lacticin 481. Here, non-standard amino acids were introduced into the structural peptide by organic synthesis, and subsequently modified *in vitro* with purified LctM to generate derivatives with superior specific activity against a target strain (Levengood et al., 2009). Notably, synthetic chemistry approaches were employed to generate hybrids of nisin and vancomycin that demonstrated a 40-fold increase in potency compared to each of the components separately (Arnusch et al., 2008). Similarly, the nisin N-terminus (1–12) was synthetically modified by the coupling of simple membrane-active lipids to create biologically active and proteolytically stable hybrids (Koopmans et al., 2015).

Regardless of these bioengineering successes, one concern that remains to be tackled is that of production. Indeed, the discovery, study and application of lantibiotics is often compromised by limited, or the absence of, production of these peptides by the native producer, a problem which is further compounded when working with bioengineered derivatives. However, a number of instances have demonstrated that quite the opposite effect can be achieved in terms of production. In the case of mutacin 1140 and nukacin ISK-1, single residue alterations did not increase specific activity but instead increased peptide production by up to fourfold (Islam et al., 2009; Chen et al., 2013). Importantly, a recent study involving synthetic biology approaches describes the development of a genetic system that facilitates significant overproduction of nisin (Kong and Lu, 2014). Although heterologous expression of lantibiotic peptides (and their bioengineered derivatives) has been demonstrated in the Gram negative host *E. coli* on several occasions (Nagao et al., 2007; Caetano et al., 2011, 2014; Shi et al., 2012; Basi-Chipalu et al., 2015), a recent study describes a multigene assembly strategy for the overexpression of the two-component lantibiotic lichenicidin in *E. coli* (Kuthning et al., 2015). Such systems may also help in attaining higher yields to simplify isolation of and improve cost-efficiency of novel derivatives that are often compromised by limited production.

A major drawback that has yet to be overcome with respect to therapeutic use is the sensitivity of lantibiotics to proteolytic cleavage by intestinal enzymes. For example, nisin, pep5 and epidermin have been shown to be susceptible to the proteases trypsin and chymotrypsin (Jarvis and Mahoney, 1969; Bierbaum et al., 1996). Bioengineering strategies could be employed to replace the residues that serve as recognition sites by these and other digestive enzymes and potentially overcome the issue of vulnerability to proteolytic breakdown in the gastrointestinal tract. Indeed, the recent discovery of the class II lantibiotic pseudomycoicidin (which was found to be resistant to trypsin) provides the perfect example for this approach. A trypsin cleavage site which is located in the conserved lipid II binding motif, is protected by the presence of at least one thioether ring structure. This was confirmed by experiments with site-directed mutant peptides where the removal of thioether forming Cys residues resulted in the establishment of protease sensitivity (Basi-Chipalu et al., 2015).

Lastly, it should be remarked that the efficacy of individual lantibiotics could be further boosted through combination with other antimicrobials or membrane-active substances. For example, nisin displayed synergistic activity with the antibiotics colistin and clarithromycin against *Pseudomonas aeruginosa* (Giacometti et al., 2000) and with ramoplanin and other non-β-lactam antibiotics against many strains of MRSA and VRE (Brumfitt et al., 2002). Similarly, nisin-ceftriaxone and nisin-cefotaxime were found to be highly synergistic against clinical isolates of *Salmonella enterica* serovar Typhimurium as evident by checkerboard test and time-kill assay (Rishi et al., 2014).

## Conclusion

Lantibiotics possess many of the attributes essential for the treatment of infections caused by multi-drug resistant bacteria and their potential for use as alternatives to traditional antibiotic therapies has been mooted for decades. While greater than 100 lantibiotic peptides have been described, not all of these have been characterized in great depth and so many may possess traits of commercial value. Indeed, as the number of microbial genome sequences has increased dramatically, an even larger collection of new lantibiotic biosynthetic gene clusters has been revealed. These clusters can be applied directly or, the information gained from their analysis, can be used indirectly to guide the bioengineering of new and existing peptide structures.

Finally, although nisin remains the only lantibiotic that is extensively exploited, its full use as a therapeutic entity has not yet been fulfilled, in part due to its low solubility and stability at physiological pH. It is thus notable that a broad range of technologies have been developed for the engineering of lantibiotics and the past decade has seen several bioengineering studies describe the generation of peptide derivatives including nisin with enhanced functionality in terms of specific activity, spectrum of activity, solubility and/or temperature and pH stability. Critically, genetic systems are in continuous development to increase yields of peptide that may aid commercial viability. The further application of these systems to enhance nisin and other lantibiotics has the potential to lead to the development of novel derivatives for therapeutic use. Additionally, bioengineering in combination with semi-synthesis will expand structural diversity still further. It is thus likely that these peptides will be only the first of many generations of bioengineered lantibiotic and lantibiotic-like peptides. Given these recent developments and the fact that several lantibiotics are currently in clinical and preclinical trials reinforces our belief that bioengineered lantibiotics can contribute to a solution to antibiotic resistance across a broad range of bacterial pathogens.

## Author Contributions

DF drafted the manuscript. PC, CH, and RR revised and approved the final manuscript.

### Conflict of Interest Statement

The authors declare that the research was conducted in the absence of any commercial or financial relationships that could be construed as a potential conflict of interest.
